# Deciphering pathophysiological mechanisms underlying cystathionine beta-synthase-deficient homocystinuria using targeted metabolomics, liver proteomics, sphingolipidomics and analysis of mitochondrial function

**DOI:** 10.1016/j.redox.2024.103222

**Published:** 2024-06-04

**Authors:** Tomas Majtan, Thomas Olsen, Jitka Sokolova, Jakub Krijt, Michaela Křížková, Tomoaki Ida, Tamás Ditrói, Hana Hansikova, Ondrej Vit, Jiri Petrak, Ladislav Kuchař, Warren D. Kruger, Péter Nagy, Takaaki Akaike, Viktor Kožich

**Affiliations:** a Department of Pharmacology, University of Fribourg, Faculty of Science and Medicine, Fribourg, 1700, Switzerland; b Department of Nutrition, Institute of Basic Medical Sciences, University of Oslo, Oslo, Norway; c Department of Pediatrics and Inherited Metabolic Disorders, Charles University-First Faculty of Medicine, Prague, 12808, Czech Republic; d Department of Pediatrics and Inherited Metabolic Disorders, General University Hospital in Prague, Prague, 12808, Czech Republic; e Department of Environmental Medicine and Molecular Toxicology, Tohoku University Graduate School of Medicine, Sendai, 980-8575, Japan; f Department of Molecular Immunology and Toxicology and the National Tumor Biology Laboratory, National Institute of Oncology, Budapest, 1122, Hungary; g Cancer Signaling and Microenvironment Program, Fox Chase Cancer Center, Philadelphia, PA, USA; h BIOCEV, First Faculty of Medicine, Charles University, 252 50, Vestec, Czech Republic; i Department of Anatomy and Histology, HUN-REN–UVMB Laboratory of Redox Biology Research Group, University of Veterinary Medicine, 1078, Budapest, Hungary; j Chemistry Institute, University of Debrecen, 4012, Debrecen, Hungary

**Keywords:** Cystathionine beta-synthase, Homocystinuria, Methionine restriction, Metabolomics, Proteomics

## Abstract

**Background:**

Cystathionine β-synthase (CBS)-deficient homocystinuria (HCU) is an inherited disorder of sulfur amino acid metabolism with varying severity and organ complications, and a limited knowledge about underlying pathophysiological processes. Here we aimed at getting an in-depth insight into disease mechanisms using a transgenic mouse model of HCU (I278T).

**Methods:**

We assessed metabolic, proteomic and sphingolipidomic changes, and mitochondrial function in tissues and body fluids of I278T mice and WT controls. Furthermore, we evaluated the efficacy of methionine-restricted diet (MRD) in I278T mice.

**Results:**

In WT mice, we observed a distinct tissue/body fluid compartmentalization of metabolites with up to six-orders of magnitude differences in concentrations among various organs. The I278T mice exhibited the anticipated metabolic imbalance with signs of an increased production of hydrogen sulfide and disturbed persulfidation of free aminothiols. HCU resulted in a significant dysregulation of liver proteome affecting biological oxidations, conjugation of compounds, and metabolism of amino acids, vitamins, cofactors and lipids. Liver sphingolipidomics indicated upregulation of the pro-proliferative sphingosine-1-phosphate signaling pathway. Liver mitochondrial function of HCU mice did not seem to be impaired compared to controls. MRD in I278T mice improved metabolic balance in all tissues and substantially reduced dysregulation of liver proteome.

**Conclusion:**

The study highlights distinct tissue compartmentalization of sulfur-related metabolites in normal mice, extensive metabolome, proteome and sphingolipidome disruptions in I278T mice, and the efficacy of MRD to alleviate some of the HCU-related biochemical abnormalities.

## Highlights

• Metabolic analysis showed distinct compartmentalization in homocystinuria (HCU).
• Liver proteome in HCU is substantially dysregulated.
• Liver lipidomics showed upregulation of sphingosine-1-phosphate signaling in HCU.
• Liver mitochondrial function was not impaired in HCU.
• Methionine restriction improved metabolic and proteomic disbalances

## Graphical abstract

**Figure:**
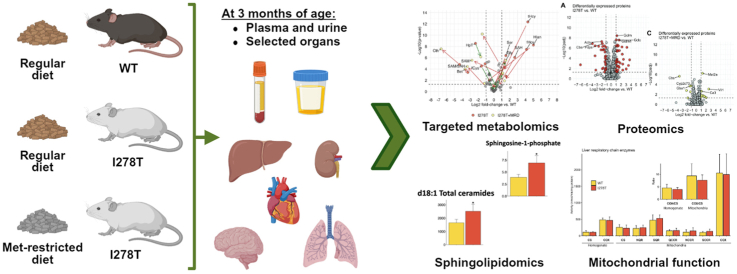

## 1 Introduction

Cystathionine β-synthase (CBS)-deficient homocystinuria (HCU) is an inborn error of sulfur amino acid metabolism inherited as an autosomal recessive trait [1,2]. Recent analysis of a cohort of 328 HCU patients showed a continuous spectrum of severity. The age of onset, diagnostic delay, and organ complications depend largely on responsiveness to pyridoxine administration [3]. Untreated HCU may affect three systems, namely vasculature (mostly thromboembolism), connective tissue (e.g., osteoporosis, marfanoid features and dislocation of ocular lenses) and nervous system (e.g., learning difficulties, intellectual disability and seizures).

Human CBS is a complex multimeric enzyme containing pyridoxal-5’-phosphate (PLP) and heme. Its activity and stability are regulated by S-adenosylmethionine (SAM) (reviewed in details in Ref. [4]). Substantially decreased CBS activity, caused by the presence of biallelic pathogenic variants in the *CBS* gene [5], blocks conversion of the dietary essential amino acid methionine (Met) to cysteine (Cys) via the transsulfuration pathway [6]. CBS deficiency thus leads to accumulation of homocysteine (Hcy), S-adenosylhomocysteine (SAH), S-adenosylmethionine (SAM) and Met, and decrease of downstream metabolites cystathionine (Cth) and cysteine (Cys). The pathophysiology of HCU is at present not completely understood. Although massive accumulation of Hcy is thought to be responsible for thromboembolism, the impact of other dysregulated metabolites, such as Met, SAM, SAH or hydrogen sulfide (H_2_S), on HCU pathophysiology is largely unknown [7,8]. Moreover, the majority of our knowledge on pathophysiology of HCU in humans is based on analyses of patients' plasma and urine, the only samples reasonably available in a clinical setting. It is plausible that tissues with different CBS expression and metabolic requirements would display a unique metabolic pattern substantially different from plasma or urine, and could respond differently to applied treatments.

Current standard of care has recently been summarized in comprehensive guidelines for the diagnosis and management of HCU [9]. The main therapeutic goal is to prevent and delay development of clinical complications in early- and late-diagnosed patients, respectively. Treatment primarily depends on pyridoxine responsiveness of the patients. The molecular mechanism of pyridoxine responsiveness in HCU is unclear [10], but most likely PLP, a catalytic cofactor of CBS, derived from pyridoxine acts as a molecular chaperone rescuing CBS folding, increasing stability and residual activity of mutant CBS. In pyridoxine responsive and extremely responsive patients, sole treatment with only pyridoxine maintains plasma total Hcy (tHcy) below 50 μmol/L. In contrast, patients with partially responsive and non-responsive form of HCU typically require dietary Met restriction (i.e., low natural protein diet with supplementation of amino acid mixtures) and/or betaine administration; in non-responsive patients, it is often difficult to maintain plasma tHcy concentrations below the therapeutic goal of less than 100 μmol/L. Despite good efficacy, poor compliance with the dietary measures and resulting compromised metabolic control substantially impacts the quality of life of HCU patients [11]. Therefore, several novel therapies are in development employing engineered human or non-human enzymes degrading Met or Hcy in the circulation or in the gut [8,12,13].

There are several mouse models of HCU, which were generated to gain understanding of HCU pathophysiology and were critical for development of emerging therapies for HCU [14]. Complete loss of CBS by gene deletion (CBS KO) in mice is associated with neonatal lethality [[15], [16], [17]]. However, this can be rescued by the introduction of a transgene expressing either a wild type human CBS or one of several pathogenic CBS variants [16,18,19]. Transgenic mice expressing the human p.I278T allele on the mouse CBS KO background (I278T) is a widely studied model. In this model, the transgene is under control of the metallothionein promoter and its expression is turned on during the neonatal period by supplying zinc in the drinking water, which rescues the neonatal lethality. The I278T mice are viable and exhibit dysregulated sulfur amino acid metabolism and phenotype similar to human HCU [18,19]. They have a shorter lifespan, decreased body weight, lower fat content, decreased bone mineralization, endothelial dysfunction, cognitive impairment, facial alopecia and damaged zonular fibers [18,[20], [21], [22], [23]]. However, in contrast to humans, I278T mice unexplainably do not respond to pyridoxine supplementation. Therefore, only dietary Met restriction, enzyme replacement therapy or gene therapy corrected plasma tHcy and total Cys (tCys) levels and ameliorated the above-mentioned phenotype associated with murine HCU [21,23,24].

The present knowledge on pathophysiology of HCU in humans is limited. However, the mouse models offer a unique opportunity for studying the dysregulation of metabolic pathways and cellular processes in tissues. Here, we examined metabolic, proteomic and sphingolipidomic changes as well as mitochondrial function in the body fluids and tissues of the I278T mouse model compared to wild type mice. In addition, we evaluated the impact of dietary Met restriction, mimicking the current standard of care for pyridoxine non-responsive HCU patients, to ameliorate the metabolic and proteomic derangements associated with murine HCU.

## 2 Materials and Methods

**Animals and study design.** The I278T mice were propagated and genotyped as described previously [18]. Breeding pairs were maintained on extruded standard diet 2920X (Envigo, USA) and water containing 25 mM ZnCl_2_ to induce transgene expression and thus rescue the homozygous I278T pups from neonatal death. After weaning at 21 days of age, mice were genotyped, provided regular drinking water and assigned into one of three groups of 8 mice. First group consisted of 4 male and 4 female wild type (WT) mice fed with standard rodent chow (Envigo 2920X, 0.5 % Met). WT mice were generated in-house and essentially were littermates of I278T mice from heterozygous breeding pairs (i.e. contained I278T transgene as well). Second group (I278T) contained homozygous 4 male and 4 female I278T mice fed with the same standard diet as WT cohort. Lastly, third group (I278T+MRD) consisted of 5 male and 3 female homozygous I278T mice provided with amino acid-defined methionine-restricted diet (MRD; Envigo TD.110591, 0.05 % Met, formulated to be isocaloric and isonitrogenous compared to the standard diet). Mice were maintained on the respective diets and regular water until reaching 3 months of age, when their blood from submandibular vein was collected into lithium heparin gel-containing tubes (Sarstedt, USA). Mice were euthanized by CO_2_ asphyxiation followed by cervical dislocation and perfused with ice-cold phosphate-buffered saline (PBS). Urine and tissues (liver, kidney, brain, heart and lung) were harvested, dissected and snap-frozen in liquid nitrogen. Tissues, urine and plasma were stored at −80 °C before further processing. Procedures involving mice were performed at the University of Colorado Anschutz Medical Campus (Aurora, CO, USA) under IACUC-approved protocol# 81. The University is an AAALAC-accredited (#00235), Public Health Service-assured (#A 3269-01) and USDA-licensed (#84-R-0059) institution. Animal experiments complied with the ARRIVE guidelines and all applicable federal and state law and institutional regulations.

### 2.1 Targeted metabolomics

***Sample preparation.*** Tissue samples were weighed and homogenized mechanically using Tissue raptor homogenizer (Qiagen) in buffer containing 1.5 % of laurylmaltoside in 100 mM TRIS pH 8.5, while tubes were held in an ice/water slush. For every 10 mg of tissue, we added 60 μl of buffer. Tissue homogenates were centrifuged at 15,000×*g*, 4 °C for 20 min. The supernatant was aliquoted and stored at −80 °C prior to metabolite analyses.

***LC-MS/MS quantification.*** Amino acids including thioethers cystathionine, lanthionine (Lan) and homolanthione (Hlan) were determined by LC-MS/MS method using commercially available Phenomenex EZ:faast kit (discontinued in 2022) for amino acid analysis as described previously [25]. Taurine (Tau), hypotaurine (HpT), choline (Chol), betaine (Bet), dimethylglycine (DMG) and S-sulfocysteine were determined by the LC-MS/MS method described in detail elsewhere [26,27]. SAM and SAH levels in plasma and tissue extracts were determined based on chromatographic separation on a Hypercarb column filled with porous graphitic carbon stationary phase using the previously published method [28]. LC-MS/MS methods were performed on a system consisting of the Agilent 1290 Infinity LC System coupled with an API 4000 triple quadrupole mass spectrometer with an electrospray ion source. The system was operated using Applied Biosystems’ Analyst software, version 1.4. Detection of analytes was carried out using positive electrospray ionization technique and selected multiple reaction monitoring.

***HPLC quantification.*** Total aminothiols and inorganic sulfur compounds were determined using Shimadzu LC-20AD HPLC system equipped with RF-20AXs fluorescence detector. Aminothiols (Cys, cysteinyl-glycine (CysGly), Hcy, GSH and γ-glutamyl-cysteine (GGCys)) were derivatized with ammonium 7-fluorobenzo-2-oxa-1,3-diazole-4-sulfonate (SBD-F) after the reduction of disulfide bonds with tris(2-carboxyethyl)phosphine (TCEP) as described previously [29]. Inorganic sulfur compounds (sulfite, thiosulfate and thiocyanate) were analyzed after derivatization with monobromobimane as described previously [26].

***Quantification of bioavailable H***_***2***_***S*.** The modified monobromobimane method was used to measure bioavailable H_2_S concentrations in plasma, as described previously [30]. Plasma or tissue homogenate (25 μl) was mixed with 66 μl of working solution (65 μl 200 mM HEPES pH 8.2 + 1 μl 100 mM monobromobimane in acetonitrile) and kept at 20 °C for 10 min before the addition of 5 μl 50 % trichloroacetic acid to stop the reaction. Precipitated proteins were removed by centrifugation at 3,000g for 5 min at RT, 10 μl of the supernatants were injected onto the HPLC column. The derivatized product (sulfide-dibimane) was separated on a Phenomenex Luna C18(2) 200 × 4.6 mm 3 μm column using a linear gradient elution with 0.1 % trifluoroacetic acid/H_2_O (A) and 0.1 % trifluoroacetic acid/acetonitrile (B) at a flow rate of 1 ml/min: 0 min, 15 % B; 3 min, 35 % B; 9 min, 35 % B; 11 min, 90 % B; 12 min, 90 % B; 13 min, 15 % B, 15 min 15 % B. Products were detected using fluorescence detector (390 nm excitation, 475 nm emission) and quantified.

***Quantification of inorganic and low-molecular-weight persulfides.*** Quantitative analysis of inorganic and low-molecular-weight persulfides was carried out using LC-ESI-MS/MS (LCMS-8060NX) with beta-(4-hydroxyphenyl)ethyl iodoacetamide (HPE-IAM). Briefly, the samples were reacted with HPE-IAM at 37 °C for 20 min. Reaction mixtures were then diluted with 0.1 % formic acid containing known amounts of isotope-labelled internal standards, followed by the measurement via LC-ESI-MS/MS essentially as described previously [31].

***Data analysis.*** Differences in sulfur-containing and related metabolites in tissues were compared for I278T vs. WT and I278T+MRD vs. WT in separate linear regression models with the log_2_-transformed metabolite concentration as the outcome and group as the predictor. For visualization purposes, all metabolites were log_2_-transformed before analysis. The resulting β-coefficients from the regression models thus represents the log_2_-fold changes (Log_2_FCs) between groups. Results are presented as volcano plots with Log_2_FCs on the x-axis and the inverse of the Log_10_p-value on the y-axis to indicate statistical significance. Regular fold changes as presented in the text and Table 1 were calculated as 2^Log2FC^. To identify metabolic signatures that may aid in classifying I278T and I278T+MRD, we performed principal component analysis (PCA). All metabolites were log_2_-transformed and centered before analysis, and the PCA and factor loading extraction were performed using the *prcomp()* function in R. For metabolites where less than 50 % of the sample had missing values, these were imputed with the sample mean prior to the PCA.

**Table 1:** Fold-change (p-values) for I278T and I278T + methionine restricted diet vs. wild-type^a^^,^^b^.

	Plasma		Urine		Liver		Kidney		Heart		Brain		Lung	
	I278T	I278T + MRD	I278T	I278T + MRD	I278T	I278T + MRD	I278T	I278T + MRD	I278T	I278T + MRD	I278T	I278T + MRD	I278T	I278T + MRD
*Methionine cycle and transsulfuration to cysteine*														
Met	1.27 (0.14)	**0.49 (< 0.001)**	1.5 (0.009)	**0.24 (< 0.001)**	1.62 (<0.001)	**0.36 (< 0.001)**	1.22 (<0.001)	0.91 (0.057)	1.26 (0.06)	0.61 (<0.001)	1.39 (0.027)	0.75 (0.051)	1.57 (0.002)	1.1 (0.44)
SAM	–	–	–	–	**2.93 (0.002)**	**0.15 (< 0.001)**	–	–	–	–	1.41 (0.006)	1.47 (0.002)	–	–
SAH	–	–	–	–	**6.19 (< 0.001)**	1.49 (0.12)	–	–	–	–	**17.7 (< 0.001)**	**2.8 (< 0.001)**	–	–
SAM/SAH	–	–	–	–	**0.47 (0.11)**	**0.10 (< 0.001)**	–	–	–	–	**0.08 (< 0.001)**	0.53 (0.003)	–	–
Sar	**2.57 (< 0.001)**	0.7 (0.069)	**2.23 (0.003)**	0.56 (0.027)	2.01 (0.076)	1.65 (0.19)	**3.45 (< 0.001)**	0.90 (0.43)	**3.64 (< 0.001)**	**0.46 (0.016)**	**2.74 (< 0.001)**	–	**3.87 (< 0.001)**	**0.36 (< 0.001)**
tHcy	**62.3 (< 0.001)**	**13.5 (< 0.001)**	**109 (< 0.001)**	**8.81 (< 0.001)**	**19.7 (< 0.001)**	**2.18 (< 0.001)**	**18.4 (< 0.001)**	**2.67 (< 0.001)**	**8.17 (< 0.001)**	1.98 (<0.001)	**7.99 (< 0.001)**	1.68 (0.005)	**40.5 (< 0.001)**	**4.1 (< 0.001)**
fHcy	**148 (< 0.001)**	**12.3 (< 0.001)**			**17.4 (< 0.001)**	**2.53 (0.012)**	–	–	–	–	–	–	–	–
Hlan	**22.7 (< 0.001)**	**12.6 (< 0.001)**	**29.8 (< 0.001)**	**23.1 (< 0.001)**	**32.2 (< 0.001)**	**0.44 (0.035)**	**28.5 (< 0.001)**	**22.9 (< 0.001)**	**13.6 (< 0.001)**	**9.14 (< 0.001)**	**53.3 (< 0.001)**	**6.24 (< 0.001)**	**18.9 (< 0.001)**	**17.9 (< 0.001)**
Cth	**0.46 (0.1)**	0.07 (<0.001)	**0.32 (0.006)**	**0.04 (< 0.001)**	**0.05 (< 0.001)**	**0.01 (< 0.001)**	0.69 (0.22)	**0.16 (< 0.001)**	1.76 (0.001)	0.90 (0.5)	**0.17 (0.002)**	**0.11 (< 0.001)**	0.59 (0.046)	**0.27 (< 0.001)**
tCys	**0.34 (< 0.001)**	0.87 (0.25)	0.95 (0.64)	**0.79 (0.034)**	**2.57 (< 0.001)**	1.5 (0.021)	1.02 (0.73)	1.2 (0.008)	1.11 (0.74)	1.85 (0.055)	0.92 (0.28)	0.88 (0.12)	1.09 (0.56)	1.06 (0.71)
fCys	**0.44 (< 0.001)**	0.93 (0.49)			**0.34 (< 0.001)**	1.07 (0.76)	–	–	–	–	–	–	–	–
Lan	**0.48 (0.001)**	**0.43 (< 0.001)**	**0.39 (< 0.001)**	**0.19 (< 0.001)**	0.58 (0.047)	0.54 (0.029)	0.51 (<0.001)	**0.47 (< 0.001)**	0.87 (0.006)	0.88 (0.01)	**0.26 (< 0.001)**	**0.25 (< 0.001)**	1.18 (0.18)	1.13 (0.33)
*Homocysteine remethylation and one-carbon metabolism*														
Chol	–	–	–	–	1.19 (0.36)	**2.08 (< 0.001)**	–	–	–	–	0.84 (0.04)	1 (0.97)	–	–
Bet	–	–	–	–	**0.10 (< 0.001)**	**0.08 (< 0.001)**	–	–	–	–	**0.48 (0.005)**	0.85 (0.49)	–	–
DMG	–	–	–	–	1.71 (0.16)	0.81 (0.56)	–	–	–	–	1.22 (0.52)	0.61 (0.11)	–	–
Ser	1.04 (0.85)	**2.53 (< 0.001)**	1.39 (0.014)	1.98 (<0.001)	**2.86 (< 0.001)**	0.91 (0.54)	1.13 (0.046)	1.29 (<0.001)	0.88 (0.4)	**2.06 (< 0.001)**	0.79 (0.02)	1.41 (0.001)	1.11 (0.38)	**2.28 (< 0.001)**
Gly	**0.80 (0.32)**	**4.61 (< 0.001)**	1.33 (0.074)	2.88 (<0.001)	0.99 (0.97)	**2.83 (< 0.001)**	1.11 (0.3)	1.29 (0.015)	0.98 (0.9)	**2.27 (< 0.001)**	0.98 (0.86)	1.07 (0.56)	1.04 (0.6)	**2.04 (< 0.001)**
*Glutathione-related metabolites*														
tGSH	1.29 (0.13)	0.89 (0.42)	1.24 (0.56)	1.37 (0.41)	**0.46 (< 0.001)**	1.23 (0.14)	0.94 (0.56)	1.49 (0.002)	1 (1)	0.81 (0.006)	0.86 (0.15)	0.64 (<0.001)	1.09 (0.46)	1.38 (0.011)
fGSH	**2.22 (< 0.001)**	0.88 (0.42)	–	–	0.66 (<0.001)	1.17 (0.15)	–	–	–	–	–	–	–	–
tGGCys	0.73 (0.031)	0.83 (0.14)	1.68 (0.13)	1.28 (0.48)	0.68 (0.012)	0.75 (0.056)	1.99 (0.002)	**4.31 (< 0.001)**	–	–	0.83 (0.35)	0.84 (0.38)	–	–
tCysGly	**0.20 (< 0.001)**	0.62 (0.002)	1.63 (0.063)	0.98 (0.95)	0.80 (0.009)	1.2 (0.029)	0.84 (0.27)	0.84 (0.29)	1.06 (0.66)	1.5 (0.008)	0.85 (0.22)	1.17 (0.24)	0.83 (0.18)	1.24 (0.13)
*Taurine-related metabolites*														
HpT	**2.14 (< 0.001)**	1.16 (0.44)	0.86 (0.41)	0.74 (0.12)	**0.20 (< 0.001)**	**0.32 (< 0.001)**	**2.11 (< 0.001)**	0.90 (0.41)	1.27 (0.006)	0.98 (0.82)	1.15 (0.24)	0.62 (<0.001)	**2.17 (0.006)**	**2.29 (0.004)**
Tau	1.07 (0.65)	0.91 (0.54)	0.54 (0.002)	0.39 (<0.001)	0.79 (0.23)	0.68 (0.055)	1.15 (0.071)	0.94 (0.42)	1 (0.98)	1 (0.96)	1.04 (0.75)	0.88 (0.25)	1.37 (0.001)	1.37 (0.001)
*Hydrogen sulfide and persulfidation*														
H_2_S (MBB)	0.98 (0.91)	**0.40 (< 0.001)**	–	–	1.32 (0.063)	1.1 (0.51)	0.85 (0.16)	0.86 (0.21)	–	–	0.72 (0.063)	0.74 (0.092)	–	–
HS^−^ (HPE-IAM)	0.68 (<0.001)	0.68 (<0.001)	–	–	1.22 (0.18)	1.15 (0.33)	–	–	–	–	–	–	–	–
HSS^−^	**0.47 (0.046)**	0.52 (0.083)	–	–	1.76 (<0.001)	1.03 (0.81)	–	–	–	–	–	–	–	–
HSSS^−^			–	–	1.03 (0.76)	0.89 (0.23)	–	–	–	–	–	–	–	–
SO_3_^2-^	1.46 (0.026)	1.4 (0.039)	1.95 (0.002)	0.82 (0.3)	0.55 (<0.001)	0.66 (<0.001)	0.64 (0.082)	0.68 (0.12)	1.12 (0.53)	**0.50 (< 0.001)**	0.75 (0.034)	**0.36 (< 0.001)**	1.9 (0.002)	0.94 (0.74)
S_2_O_3_^2−^ (MBB)	1.83 (0.039)	0.69 (0.18)	3.54 (0.061)	0.59 (0.41)	0.95 (0.77)	1.97 (<0.001)	1.79 (<0.001)	0.97 (0.84)	1.17 (0.33)	0.85 (0.31)	1.74 (0.014)	1.28 (0.25)	**2.08 (< 0.001)**	1.27 (0.19)
HS_2_O_3_^−^ (HPE-IAM)	1.27 (0.37)	0.43 (0.004)	–	–	0.98 (0.96)	0.79 (0.49)	–	–	–	–	–	–	–	–
SCN^−^	0.72 (0.61)	0.90 (0.87)	1.00 (1)	1.25 (0.78)	1.83 (0.54)	4.3 (0.19)	1.24 (0.43)	1.08 (0.78)	1.15 (0.69)	0.81 (0.56)	0.88 (0.45)	1.42 (0.048)	1.26 (0.23)	**2.27 (< 0.001)**
GSSH	0.93 (0.79)	0.41 (0.004)	–	–	1.45 (0.048)	1.23 (0.26)	–	–	–	–	–	–	–	–
HcySSH	72 (n/a)	9.9 (n/a)			1.4 (n/a)									
CysSSH	**0.36 (< 0.001)**	**0.50 (< 0.001)**	–	–	1.99 (0.026)	0.66 (0.16)	–	–	–	–	–	–	–	–
*Other amino acids*														
Ala	0.91 (0.72)	1.31 (0.27)	1.12 (0.24)	1.34 (0.005)	0.97 (0.9)	1.16 (0.46)	1.18 (0.033)	1.07 (0.37)	0.85 (0.076)	0.89 (0.19)	0.94 (0.44)	0.91 (0.25)	0.96 (0.83)	1.36 (0.12)
Val	1.02 (0.89)	1.01 (0.95)	1.08 (0.41)	1.14 (0.17)	1.28 (0.019)	0.70 (0.001)	1.13 (0.058)	0.89 (0.071)	0.88 (0.32)	1.04 (0.77)	1.03 (0.72)	1.18 (0.076)	1.22 (0.18)	1.3 (0.088)
Phe	1.02 (0.89)	0.93 (0.57)	1.06 (0.52)	0.83 (0.053)	**2.53 (< 0.001)**	0.84 (0.34)	1.11 (0.032)	0.94 (0.16)	1.01 (0.82)	0.81 (0.001)	1.08 (0.38)	1.05 (0.62)	1.25 (0.026)	1.23 (0.035)
Tyr	–	–	–	–	1.85 (<0.001)	0.63 (0.001)	1.17 (0.005)	1.01 (0.89)	1.1 (0.35)	0.94 (0.52)	1.24 (0.046)	1.24 (0.05)	1.33 (0.016)	1.33 (0.017)
Leu	1.05 (0.81)	0.73 (0.11)	1.14 (0.45)	0.73 (0.069)	1.5 (<0.001)	0.64 (<0.001)	1.15 (0.012)	0.87 (0.011)	1.02 (0.81)	0.81 (0.042)	1.08 (0.37)	0.89 (0.18)	1.16 (0.23)	1.12 (0.37)
Ile	1.04 (0.86)	1.08 (0.71)	0.80 (0.3)	1.14 (0.53)	1.53 (<0.001)	0.76 (0.008)	1.11 (0.17)	0.83 (0.015)	0.91 (0.47)	1.07 (0.59)	0.97 (0.66)	1.08 (0.35)	1.11 (0.41)	1.43 (0.009)
His	–	–	–	–	0.85 (0.31)	0.66 (0.017)	1.19 (0.049)	1.24 (0.021)	0.76 (0.052)	**0.38 (< 0.001)**	1.11 (0.4)	0.87 (0.27)	1.3 (0.057)	0.96 (0.77)

a Fold-changes and p-values were derived from separate linear regression models for each metabolite including the grouping variable with wild-type (WT) as the reference group. See statistical methods for further details.

b Abbreviations: Ala, alanine; Bet, betaine; Chol, choline; Cth, cystathionine; CySSH, cystine; DMG, dimethylglycine; fCys, free cysteine; fGSH, free glutathione; fHcy, free homocysteine; GSSH, glutathione persulfide; Gly, glycine; H_2_S, bioavailable sulfide; His, histidine; Hlan, homolanthionine; HpT, hypotaurine; HS^−^, free sulfide; HS_2_O_3_^−^, hydrogen thiosulfate; HSS^−^, H_2_S persulfide; HSSS^−^, H_2_S trisulfide; Ile, isoleucine; Lan, lanthionine; Leu, leucine; Met, methionine; Phe, phenylalanine; SAH, S-adenosylhomocyteine; SAM, S-adenosylmethionine; Sar, sarcosine; SCN^−^, thiocyanate; Ser, serine; SO_3_^2−^, sulfite; S_2_O_3_^2−^, thiosulfate; Tau, taurine; tCys, total cysteine; tCysGly, total cysteinylglycine; tGGCys, total gamma-glutamylcysteine; tGSH, total glutathione; tHcy, total homocysteine; Tyr, tyrosine; Val, valine.

### 2.2 Proteomics

***Sample preparation.*** Liver samples (n = 4 for each cohort) previously pulverized in liquid nitrogen were lysed in 5 % sodium deoxycholate in 100 mM triethylammonium bicarbonate, incubated 15 min at room temperature and sonicated for 3 min with 15/5 s sonication/pause cycles (Q125 Sonicator, QSonica). Insoluble material was separated by centrifugation at 10,000×*g* for 10 min. Protein concentration in the clarified supernatants was determined by using a BCA assay (Merck). Individual samples containing 120 μg of protein material were diluted with 100 mM triethylammonium bicarbonate to final volume of 100 μl, then were reduced by a final 10 mM TCEP at 55 °C for 1 h and alkylated by adding iodoacetamide to a final 20 mM concentration and incubating at room temperature in the dark for 30 min. The alkylated proteins were precipitated overnight at −20 °C by 600 μl of pre-chilled acetone. Precipitate was harvested by centrifugation at 15,000×*g*, 4 °C for 15 min.

***Protein digestion and TMT labelling.*** Dried protein pellets were resuspended in 100 μl of 100 mM triethylammonium bicarbonate with trypsin (Promega, 2.5 μg per sample) overnight at 37 °C. The twelve TMT labels from the TMTpro 16plex Label Reagent Set (ThermoScientific) were assigned to the sample quadruplicates as follows: I278T samples to labels 126C, 127 N, 127C and 128 N, WT samples to labels 128C, 129 N, 129C and 130 N, and the I278T+MRD samples to labels 130C, 131 N, 131C and 132 N. Labelling reaction lasted 1 h at room temperature and was terminated by addition of 5 μl of 5 % hydroxylamine and further 15 min incubation at room temperature. All labelled samples were combined into a single tube, desalted using an OptiTrap column (Optimize Technologies) and dried using a SpeedVac vacuum concentrator (ThermoScientific).

***2D-LC-MS/MS analysis.*** The TMT-labelled peptide sample was first fractionated using Pierce High pH Reversed-Phase Peptide Fractionation Kit (ThermoScientific) according to the manufacturers’ instructions into eight fractions. Each fraction was resuspended in 1 % trifluoroacetic acid in 2 % acetonitrile and roughly 1.5 μg of peptide was injected onto nanoHPLC Dionex Ultimate 3000RS connected to Thermo Orbitrap Fusion. Thermo PepMap Trap column was used for peptide pre-concentration and the separation was done on 50 cm EASY Spray Column (ThermoScientific) using 180 min gradient from 2 % A to 35 % B (A: 0.1 % formic acid, B: 0.1 % formic acid in acetonitrile). Cycle time was set to 4 s. The MS^2^ spectra for identification were measured in an ion trap with CID fragmentation, 60 ms maximum injection time, and the MS^3^ spectra were measured in an Orbitrap with HCD fragmentation using the SPS function, 118 ms maximum injection time, 10 precursors for synchronous precursors selection feature. The LC-MS/MS analysis was performed twice for each fraction.

***Raw LC-MS data processing.*** Raw data from LC-MS/MS analyses of all fractions were searched together with Proteome Discoverer 2.5 with Sequest search engine. The method was based on a predefined workflow for SPS MS^3^ isobaric quantification with the batch specific correction parameters for the used TMT 16plex labels. Searches were performed using a database of Human – Uniprot reviewed (Release 2022_11) along with common contaminants database. Dynamic modifications were set as follows: oxidation (+15.995 Da (M)), protein N-terminal acetylation (+42.011 Da), Met-loss (−131.040 Da (M)) and Met-loss+acetylation (−89.030 Da (M)). Static Modifications: Carbamidomethyl (+57.021 Da (C)), TMT6plex (+229.163 Da (K)), peptide N-terminus TMT6plex (+229.163 Da). FDR was set up at 0.01 for both peptide and protein using Percolator. For reporter ions detection 20 ppm tolerance was set with most confident centroid peak integration. Adjusted p-values were calculated by the software using the Benjamini-Hochberg approach. The mass spectrometry proteomics data have been deposited to the ProteomeXchange Consortium via the PRIDE partner repository with the dataset identifier 10.6019/PXD049417.

***Data analysis.*** Only the proteins identified with at least two unique peptides, and all TMT ratios determined across all the cohorts and replicates were included into the quantitative evaluation. For exploratory pathway analyses, all differentially expressed genes with an adjusted p-value <0.05 were included. Adjusted p-values were calculated using the Benjamini-Hochberg approach. Enrichment analyses were performed using the *enrichPathway()* function from the ReactomePA package in R. The enrichment analyses were performed against a background consisting of the n = 3986 proteins present in the custom proteomic dataset.

### 2.3 Sphingolipidomics

Sphingolipids were measured, quantified and analyzed by previously published methods. Specifically, 10 % tissue homogenate for analysis of sphingosine-1-phosphate, ceramides and sphingosines was processed by a previously published method [32], while sphingomyelin and ceramides monohexosides were determined as described previously [33].

### 2.4 Mitochondrial function

***Homogenization and isolation of the mitochondrial fraction.*** Frozen liver sample (10 % w/v) was homogenized in ice-cold STEA buffer (10 mM TRIS-HCl pH7.4, 250 mM sucrose, 1 mM EDTA, 1 % Protease Inhibitor Cocktail) by using a Potter–Elvehjem homogenizer. Resulting homogenate was used for activity measurement of total cytochrome *c* oxidase (COX) and citrate synthase (CS) and for determination of total coenzyme Q (CoQ) content. The postnuclear supernatant (PNS) was prepared from the homogenate by centrifugation at 600×*g*, 4 °C for 10 min. The PNS was filtered through a nylon mesh. The mitochondria were pelleted by centrifugation of the PNS at 10,000×*g*, 4 °C for 10 min, washed with STEA buffer and finally resuspended in STEA buffer to a protein concentration of approximately 20 mg/ml. Aliquots of the isolated mitochondria were used for the enzyme activity determination.

***Activities of mitochondrial enzymes.*** NADH ubiquinone reductase (NQR, complex I), succinate ubiquinone reductase (SQR, complex II), ubiquinol cytochrome *c* oxidoreductase (QCCR, complex III), cytochrome *c* oxidase (COX, complex IV), NADH cytochrome *c* reductase (NCCR, complex I+III), and succinate cytochrome *c* reductase (SCCR, complex II+III) were measured spectrophotometrically using Shimadzu 2401 UV-VIS spectrophotometer at 37 °C in isolated mitochondria from the frozen tissue according to the method used by Rustin et al. [34], while activity of citrate synthase (CS) was measured according to the method described by Srere et al. [35].

***Total CoQ content.*** The total CoQ content (represented as Q9 fraction) in liver homogenate was determined using HPLC with UV detection at 275 nm according to the method of Mosca et al. [36] with minor modifications described in Křížová et al. [37]. The results were expressed as pmol CoQ/mg protein.

***Protein content.*** The total protein concentration of the homogenates for all mitochondrial analyses was determined by the Lowry method [38].

## 3 Results

### 3.1 Targeted metabolomics

**Fig. 1:**
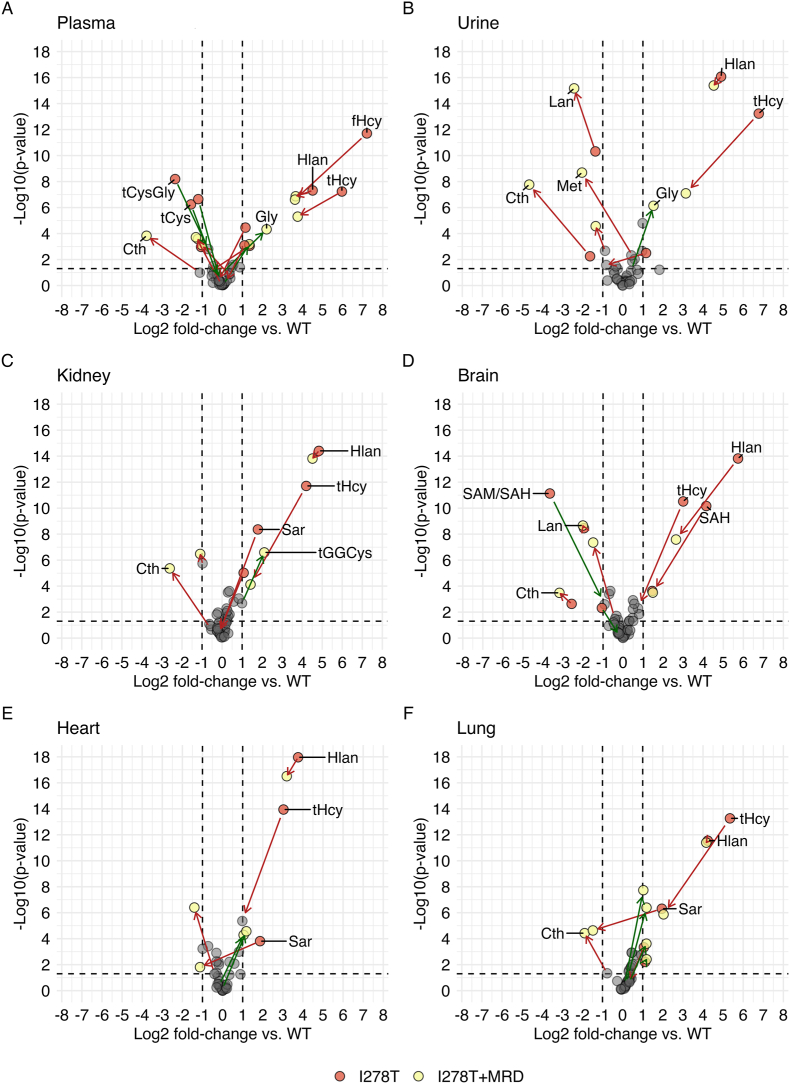
**Targeted metabolomics of plasma, urine and selected tissues.** Sulfur amino acid metabolites and related compounds were determined as described in the Materials and Methods in plasma (A), urine (B) and tissue homogenates from kidney (C), brain (D), heart (E) and lung (F). Plots show Log_2_FCs of I278T cohort on standard rodent diet (red circles) and I278T+MRD cohort (yellow circles) compared to WT controls on standard rodent diet. For values lower than the threshold (±1 of log_2_FC vs. WT), grey circles were used for both groups. Green and red arrows indicate increase and decrease of the change, respectively. Concentrations of all individual metabolites are shown in Supplementary Table 1.

**Fig. 2:**
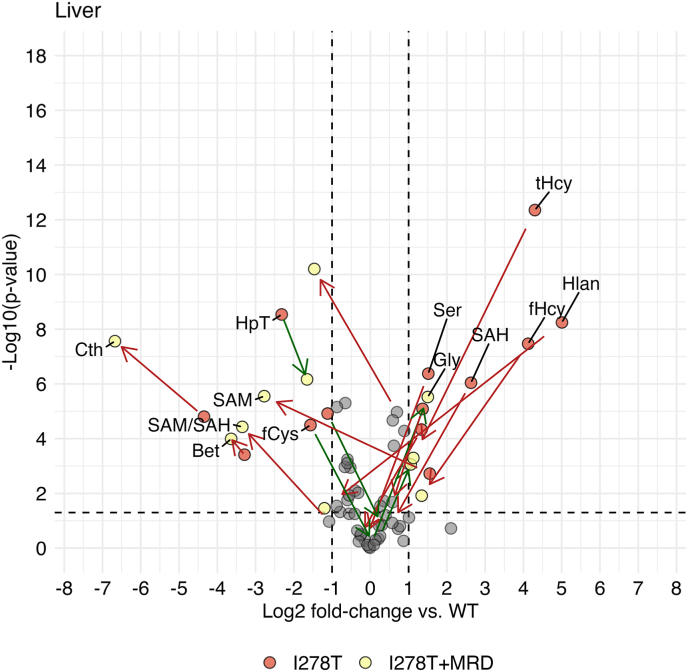
**Targeted liver metabolomics.** Liver sulfur amino acid metabolites and related compounds were determined as described in the Materials and Methods. Plots show Log_2_FCs of I278T group on standard rodent diet (red circles) and I278T MRD group (yellow circles) compared to WT group on standard rodent diet. For values lower than the threshold (±1 of log_2_FC vs. WT) grey circles were used for both groups. Green and red arrows show increase and decrease of the change, respectively. Concentrations of all individual metabolites are shown in Supplementary Table 1.

Fig. 1 shows differences in the quantified sulfur-containing and sulfur metabolism-related compounds in plasma (Fig. 1A), urine (Fig. 1B), kidney (Fig. 1C), brain (Fig. 1D), heart (Fig. 1E) and lung (Fig. 1F) as Log_2_FCs in I278T versus WT and I278T+MRD versus WT. Similar presentation of changes in liver as the main metabolic organ is shown separately in Fig. 2. Table 1 shows numerical fold-changes for all analytes and tissues calculated by back-transformation, and adjusted p-values used for graphical presentation in Fig. 1, Fig. 2. Supplementary Table 1 shows concentrations of each individual metabolite in all three mouse cohorts. In the following paragraphs we discuss the selected results relevant for HCU pathophysiology and treatment efficacy.

**Met**, an essential amino acid and precursor of Hcy, was 1.2–1.6-fold increased in body fluids and tissues, except of plasma and heart, in I278T mice compared to the WT cohort. The treated I278T+MRD cohort exhibited an anticipated decrease of Met to 0.5-, 0.2-, 0.4- and 0.6-fold in plasma, urine, liver and heart, respectively.

**SAM and SAH** were determined only in liver and brain, the key organs affected in murine HCU [22,23]. Compared to WT controls, the I278T mice exhibited 2.9-fold and 1.4-fold increased **SAM** in liver and brain, respectively. Similarly, **SAH** was 6.2- and 17.8-fold higher in liver and brain, respectively. In the I278T+MRD cohort, liver SAM decreased to 0.2-fold of WT mice, while brain SAM remained 1.5-fold elevated. SAH in brain dropped down to 2.8-fold compared to WT mice. **Sar** is generally recognized as a marker of enhanced removal of SAM via hepatic glycine-N-methyltransferase (GNMT), but can also be produced from dimethylglycine. Sar was 2.2–3.9-fold elevated in all body fluids and tissues except of liver of I278T mice. The diet caused Sar decrease in all compartments of I278T+MRD cohort except of liver.

As expected in HCU, **tHcy** was massively elevated in all body fluids and tissues of I278T compared to WT mice: from ∼8-fold in brain and heart, ∼19-fold in liver and kidney, 62-fold in plasma up to a 109-fold in urine. In addition, **free Hcy** was also elevated by 148- and 17-fold in plasma and liver, respectively, using a different method (see section on persulfidation). These results clearly indicate that body fluids, such as plasma and urine, serve as sink reservoirs for Hcy disposal from tissues. Treatment substantially decreased Hcy production and subsequent accumulation in I278T+MRD mice by an order of magnitude in urine, lung, liver and kidney and ∼4-fold in plasma, brain and heart compared to I278T mice on a standard diet, although these levels still remained 1.7–13.5-fold higher than in WT mice. **Hlan** is a thioether produced from the excess of Hcy by cystathionine γ-lyase (CGL) [39]. Unsurprisingly, Hlan was massively elevated across all tissues and body fluids of I278T mice compared to WT, particularly in liver and brain (∼32 and ∼53-fold increase, respectively). Due to its stability, lack of binding to proteins and global increase in all tissues and body fluids, Hlan may possibly serve as an alternative biomarker of accumulation of Hcy [40]. Met restriction substantially decreased Hlan levels in the I278T+MRD cohort to 0.4- and 6.2-fold of WT levels in the liver and brain, respectively, while changes in body fluids and other tissues were much less pronounced.

The canonical pathway to remove excess of Hcy is its conversion to Cys via the transsulfuration pathway; its first step being catalyzed by CBS. As expected in CBS-deficient HCU, concentrations of CBS product **Cth** were substantially decreased in plasma, urine and tissues of I278T mice to 0.7–0.05-fold with exception of heart where Cth was increased 1.8-fold compared to WT mice. Met restriction further exacerbated lack of Cth in all tested samples of I278T+MRD mice. **Lan**, a thioether similar to Cth, which formation is also catalyzed by CBS [40,41], was found similarly decreased in I278T mice to 0.3–0.9-fold compared to WT controls with the exception of lung, which remained normal. Met restriction did not further change these levels in the I278T+MRD cohort.

Other canonical pathway to metabolize Hcy is its remethylation back to Met via the ubiquitously expressed methionine synthase. An alternative catabolic pathway of Hcy remethylation utilizes the liver-dependent enzyme betaine-homocysteine methyltransferase (BHMT) [42]. We quantified **betaine**, a co-substrate of BHMT serving as a methyl group donor, along with its precursor **choline** and the by-product **DMG** in liver and brain. Liver and brain choline remained similar in all three mouse cohorts. However, betaine was markedly depleted to 0.1- and 0.5-fold in liver and brain of in I278T mice, respectively, suggesting an increased detoxification of Hcy via the BHMT shunt, which was further indirectly supported by a small, non-significant increase of DMG. The liver of I278T+MRD cohort contained 2.1-fold more choline with persisting depletion of betaine while there was no significant difference in brain compared to WT mice.

Concentration of **tCys**, the final product of the transsulfuration pathway, in I278T mice was similar to WT mice across body fluids and tissues except of plasma with a 0.3-fold decrease and liver with a 2.6-fold increase. **Free Cys** was also decreased to 0.4-fold in plasma, but also decreased in liver to 0.3-fold of WT mice. Diet resulted in a decrease of tCys to 0.8- and 0.9-fold in urine and brain, respectively, of the I278T+MRD cohort and a persisting increase of 1.5-fold in liver with a simultaneous normalization of hepatic fCys content.

Cys is a rate limiting precursor for synthesis of GSH, the abundant cellular small molecule redox regulator [43]. **Total GSH** in tissues and body fluids of I278T mice remained similar compared to WT controls with exception of a 0.5-fold decrease in liver accompanied by a 0.7-fold decrease and 2-fold increase of **free GSH (fGSH)** in liver and plasma, respectively. Diet resulted in 0.8- and 0.6-fold decrease of tGSH in heart and brain, respectively, and 1.5- and 1.4-fold increase in kidney and lung, respectively, in the I278T+MRD cohort compared to WT mice. **Total GGCys**, an intermediate in GSH synthesis, was decreased to ∼0.7-fold in plasma and liver of I278T mice and increased 2.0-fold in kidney compared to WT cohort. Met restriction further increased tGGCys concentrations in kidney of I278T+MRD mice, possibly indicating an enhanced synthesis of GSH. **Total CysGly**, a degradation product of GSH, was decreased in plasma and liver of I278T mice to 0.2- and 0.8-fold, respectively, compared to the WT controls. Met restriction normalized liver tCysGly and increased its level in plasma to 0.6-fold compared to WT.

We also analyzed oxidative Cys catabolism by evaluating concentrations of HpT and Tau [44]. **HpT** was ∼2-fold elevated in plasma, kidney and lung and decreased to 0.2-fold in liver of I278T compared to the WT mice. Met restriction normalized HpT in plasma and kidney, but the HpT imbalance remained similar in liver and lung of I278T+MRD compared to I278T cohort. **Tau** was not significantly changed in both I278T and I278T+MRD mice compared to WT control except for urine and lung, where it was decreased to 0.4–0.5-fold and increased to ∼1.4-fold, respectively.

Furthermore, we analyzed several amino acids, such as Gly, Ser, Ala, Phe, Tyr, His, Val, Leu and Ile (Table 1). In general, analysis of changes in amino acids among cohorts and its significance is complicated likely due to a significant difference between a complete diet used for I278T and WT cohorts and amino-acid defined diet used for I278T+MRD mice.

### 3.2 Inorganic sulfur compounds and persulfides

**Fig. 3:**
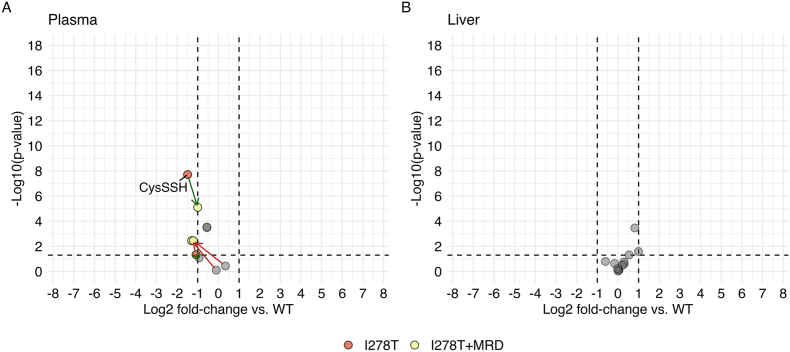
**Plasma and liver inorganic and low-molecular-weight persulfide analysis.** Free thiols and their persulfides were quantified in plasma (A) and liver (B) as described in the Materials and Methods. Plots show Log_2_FCs in I278T cohort on standard rodent diet (red circles) and I278T+MRD cohort (yellow circles) compared to WT controls on standard rodent diet. For values lower than the threshold (±1 of log_2_FC vs. WT), grey circles were used for both groups. Green and red arrows show increase and decrease of the change, respectively. Concentrations of all individual metabolites are shown in Supplementary Table 2.

In the section above, we reported a universal elevation of Hlan and decreased concentration of Lan across tissues as indirect biomarkers of increased H_2_S biosynthesis from Hcy and decreased production from Cys, respectively. To gain further insight into H_2_S homeostasis and its mitochondrial oxidation, we determined inorganic sulfur compounds and inorganic and low-molecular-weight persulfides in selected tissues using the MBB or HPE-IAM method, respectively (Table 1, Fig. 1, Fig. 3, Supplementary Tables 1 and 2). **Bioavailable H**_**2**_**S** was unchanged and decreased to 0.7-fold by the MBB and HPE-IAM approach, respectively, in plasma of I278T mice compared to WT mice. Bioavailable H_2_S was largely unchanged in the remaining samples except of the liver showing a non-significant 1.2–1.3-fold increase by both methods. **Persulfide (HSS**^**−**^**)** was decreased to 0.5-fold in plasma of I278T mice compared to the WT controls, while hepatic HSS^−^ concentrations were 1.8-fold elevated along with the detectable **trisulfide (HSSS**^**−**^**)** in livers of the I278T cohort. These data are congruent with elevated Hlan that indicated an overproduction of H_2_S from the excess of Hcy via CGL in the liver of murine HCU model. Met restriction decreased plasma bioavailable H_2_S measured by MBB method to 0.4-fold compared to WT, but, in general, had only a minor impact on other analyzed tissues. Analysis of Cys and Hcy persulfidation showed significant decrease of **Cys persulfide (CysSSH)** to 0.4-fold in plasma of I278T versus WT cohort and 2-fold increase in liver CysSSH concentration, respectively. **Hcy persulfide (HcySSH)** was detectable only in plasmas of I278T and I278T+MRD mice as well as livers of I278T mice. Met restriction normalized and slightly increased the concentration of CysSSH in plasma and liver, respectively, while levels of plasma HcySSH decreased 8-fold in I278T+MRD mice compared to I278T cohort.

The first step in the canonical H_2_S catabolism to sulfate is catalyzed by sulfide:quinone oxidoreductase (SQOR) and produces **GSH persulfide (GSSH)**. In line with the hepatic H_2_S overproduction described above, the liver GSSH concentration in I278T mice was 1.5-fold elevated compared to WT mice while plasma GSSH remained normal. Dietary restriction decreased plasma GSSH to 0.4-fold, which, however, remained elevated in liver.

**Sulfite**, an intermediate in oxidative catabolism of H_2_S, was increased 1.5-, 2.0- and 1.9-fold in plasma, urine and lung, but decreased to 0.6-fold in liver of I278T mice compared to WT controls. Met restriction resulted in a decrease of sulfite in all samples except of plasma in I278T+MRD mice, where it remained 1.4-fold elevated compared to WT controls. Analysis of thiosulfate and thiocyanate was complicated due to high analytical variability as well as sex-related differences, particularly apparent in plasma and urine samples with males producing more thiocyanate than females and females producing more thiosulfate than males. Nevertheless, excretion of **thiosulfate (S**_**2**_**O**_**3**_^**2−**^**)** as an intermediate originating from sulfite was 3.5-fold increased (with a trend for significance) in I278T mice and its concentration was also increased 1.7–2.1-fold in plasma, kidney, brain and lung compared to the WT cohort.

### 3.3 Principal component analysis and compartmentalization of metabolites

**Fig. 4:**
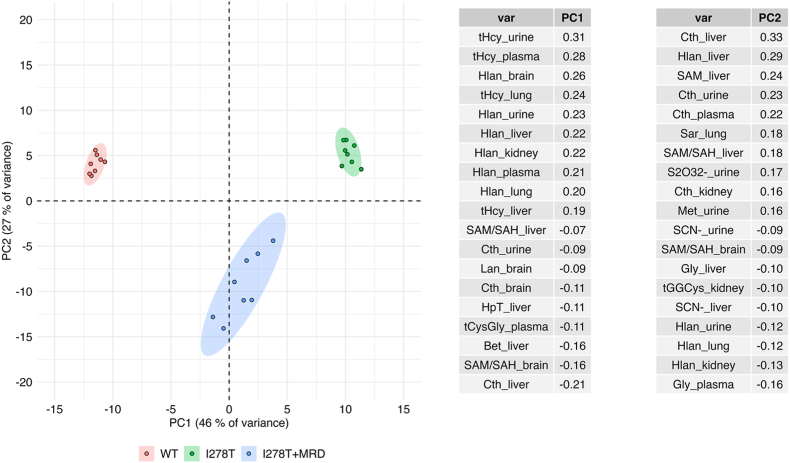
**Principal component analysis of metabolic changes.** Classification of mouse cohorts as defined by PC1 and PC2 and correlation coefficients (see Materials and Methods for details). Consistent with the HCU phenotype, PC1 correlated positively with tHcy in urine, plasma and lung, and with Hlan in all tissues. PC2 correlated positively with liver concentrations of Cth, Hlan, SAM, as well as plasma and urine Cth. Consistent with the results for the individual metabolites, I278T mice (green points/ellipse) on chow had high PC1 scores compared to I278T+MRD (blue points/ellipse) and WT cohorts (red points/ellipse). I278T+MRD cohort had higher PC1 scores but lower PC2 scores than WT mice.

To integrate all metabolic changes in body fluids and tissues, we performed **principal component analysis** of the quantified sulfur-containing and sulfur metabolism-related compounds. Fig. 4 shows clear separation between the diet groups according to principal components PC1 and PC2. PC1 explained 47 % of the variation and correlated positively with tissue concentrations of tHcy and Hlan, which is consistent with the HCU phenotype. I278T mice had higher PC1 scores compared to both I278T+MRD and WT mice (both p < 0.001). I278T+MRD mice had higher PC1 scores compared to WT mice (p < 0.001). PC2 explained 26 % of the variation and correlate positively with liver Cth, Hlan and SAM, all of which were considerably reduced in the I278T+MRD diet vs. both I278T and WT mice. Accordingly, I278T+MRD mice had lower PC2 scores than both I278T and WT mice (p < 0.001).

**Supplementary Fig. 1:**
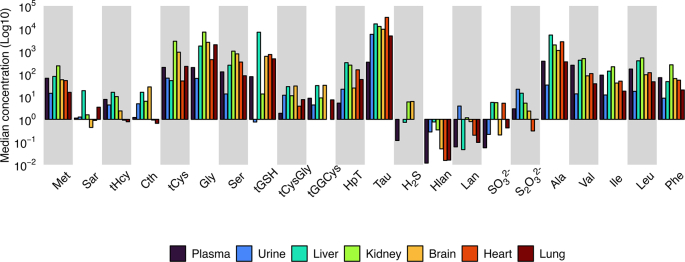
Compartmentalization of sulfur amino acid metabolites and related compounds in WT mice. Concentration of metabolites on the log_10_ scale in different body fluids (μmol/L in plasma and μmol/mmol creatinine in urine) and tissues (μmol/kg of liver, kidney, brain, heart and lung) of WT mice.

**Supplementary Fig. 2:**
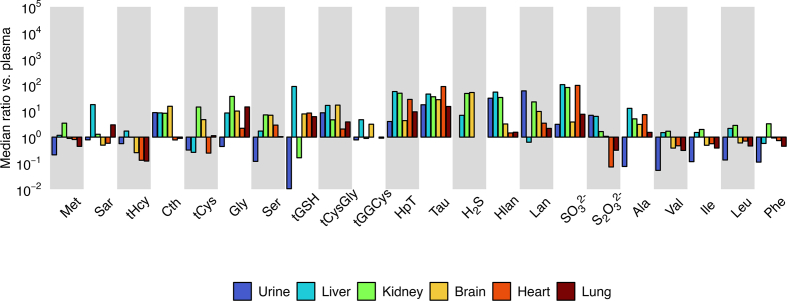
Compartmentalization of sulfur amino acid metabolites and related compounds and related metabolites. Median ratios of concentrations of metabolites in urine (μmol/mmol creatinine) and tissues (μmol/kg liver, kidney, brain, heart and lung) to plasma (μmol/L) on the log_10_ scale in WT mice.

Furthermore, quantification of the metabolites in body fluids and multiple tissues allowed us to analyze **tissue compartmentalization** of the sulfur compounds. Supplementary Fig. 1 shows median absolute concentrations of individual metabolites expressed as μmol/L of plasma and μmol/kg of tissue to enable direct comparison. There is up to six orders of magnitude difference among the metabolites. While Hlan was present in submicromolar concentrations, Tau content in tissues was in dozens millimolar concentrations. Supplementary Fig. 2 shows ratios of metabolite concentrations in the tissues compared to plasma. Liver, kidney and brain represent three organs with the highest dynamic range showing up to ∼3 orders of magnitude difference in the majority of metabolites compared to plasma. Lung and heart showed substantial changes only for the handful of metabolites with overall metabolic ratios similar to plasma.

### 3.4 Proteomics

**Fig. 5:**
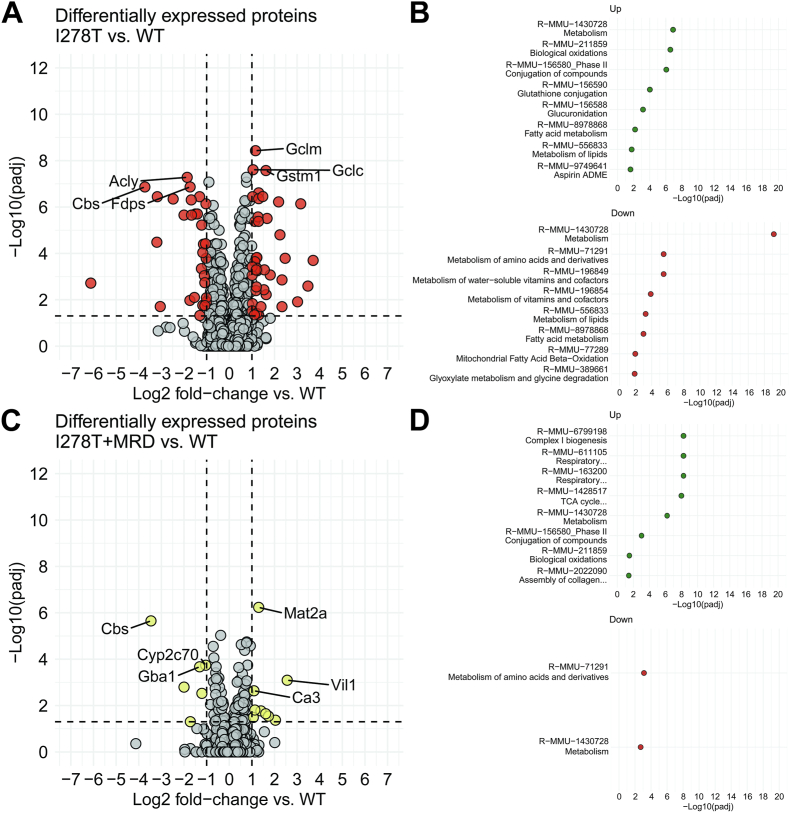
**Liver proteomics.** Liver proteome was determined as described in the Materials and Methods. Plots show Log_2_FCs in I278T mice versus WT on standard rodent diet (A) and I278T mice on MRD versus WT controls on standard rodent diet (C). Red and yellow colors indicate the most markedly and significantly differentially expressed proteins either above or below a threshold (±1 of log_2_FC vs. WT). Panels B and D show enrichment analyses for proteomics analyses shown in panels A and C, respectively. Abundances and significance values for each differentially expressed protein are shown in Supplementary Tables 3–6.

We also performed untargeted proteomics to complement the metabolomics as well as to understand changes and disruptions in cellular proteome in the key metabolic organ in HCU, liver. Out of the 5554 mouse proteins we identified, 3986 were detected with at least two distinct peptides and quantified across all 12 TMT channels, i.e. across all replicates and cohorts. We found 386 significantly differentially expressed proteins (adjusted p-value <0.05) with 212 upregulated and 174 downregulated in I278T versus WT control mice both on standard rodent chow (Fig. 5A, Supplementary Tables 3 and 4). Among the significantly enriched pathways were biological oxidations, conjugation of compounds including glutathione conjugation and glucuronidation (up), and metabolism of amino acids, vitamins, cofactors and lipids including fatty acids (down) (Fig. 5B).

Met restriction in I278T mice substantially reduced dysregulation of liver proteome as indicated by only 96 significantly differentially expressed proteins with 58 upregulated and 38 downregulated in I278T+MRD versus WT controls (Fig. 5C–Supplementary Tables 5 and 6). Among the significantly enriched pathways were complex I biogenesis and respiratory electron transport (up), and metabolism of amino acids (down) (Fig. 5D). Full proteomics data are available via ProteomeXchange with identifier PXD049417.

### 3.5 Sphingolipidomics

**Fig. 6:**
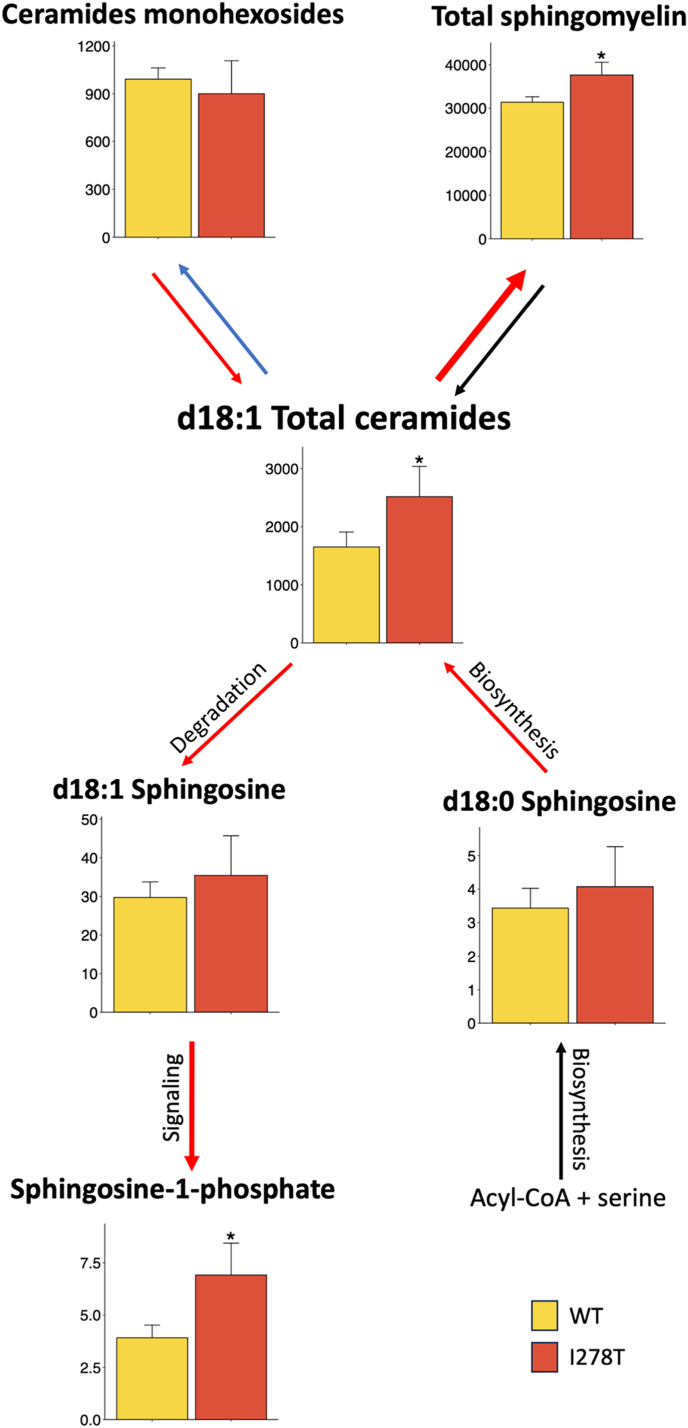
**Liver sphingolipidomics.** Selected sphingolipids with focus on ceramides were quantified by LC and FIA coupled to ESI-MS/MS as described in the Materials and Methods in liver homogenates of I278T and WT mice on a standard diet (n = 4 M+4F each cohort). Males and females were pooled together and asterisk denote significant change in I278T compared to WT controls and were derived from linear regression models.

Dysregulated liver lipid metabolism in HCU has been previously demonstrated in mouse models of HCU as well as in liver biopsies from HCU patients [22,45,46]. To gain more insight into dysregulation of lipid signal transduction pathways in HCU, we carried out a focused analysis of liver sphingolipids in I278T versus WT mice on standard diet (Fig. 6; Supplementary Table 7). Ceramides, the central molecules in sphingolipids metabolism, were significantly 1.5-fold elevated in I278T versus WT mice. It is unclear, however, what is the source of elevated ceramides as sphingosines d18:0 and d18:1 as indicators of ceramides biosynthesis and degradation, respectively, did not show significant differences. Overall increase of ceramides in I278T mice was subsequently reflected in a significant 1.2-fold increase of a representative phospho-sphingolipid sphingomyelin as well as 1.8-fold increased concentrations of sphingosine-1-phosphate (S1P), a major pro-proliferative and pro-survival signaling molecule. Taken together, our liver sphingolipidomics indicate upregulation of sphingomyelin-ceramide-S1P signaling pathway in I278T mice.

### 3.6 Mitochondrial function

**Fig. 7:**
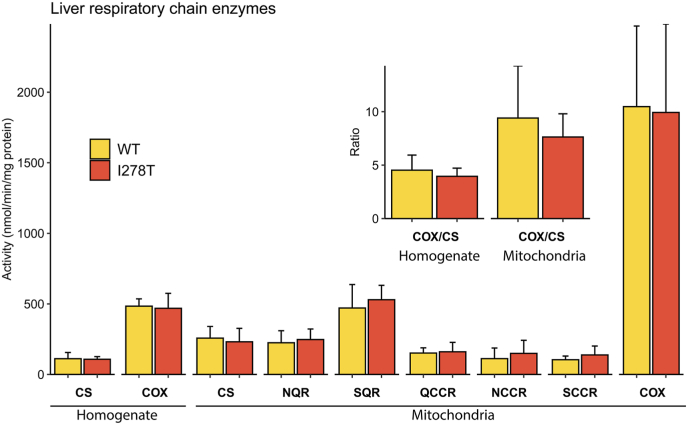
**Mitochondrial function.** Activities of the selected enzymes involved in mitochondrial oxidative phosphorylation were determined in liver homogenates of I278T and WT mice on standard diet (n = 4 M+4F each cohort) and in isolated mitochondrial fractions as described in the Materials and Methods. Quantified enzymatic activities: citrate synthase (CS), cytochrome *c* oxidase (COX), citrate synthase (CS), NADH quinone oxidoreductase (NQR), succinate quinone reductase (SQR), quinol cytochrome *c* reductase (QCCR), NADH cytochrome *c* oxidase (NCCR) and succinate cytochrome *c* oxidase (SCCR). Inset shows COX/CS ratios in liver homogenate and mitochondrial fractions.

Swollen, disorganized mitochondria were observed by electron microscopy in liver biopsies of HCU patients as well as in liver of CBS knockout mouse model of HCU [17,47,48]. Therefore, we investigated whether such structural and morphological changes come with functional consequences. Unexpectedly, activities of multiple enzymes involved in mitochondrial respiration and oxidative phosphorylation were not significantly altered in liver homogenates of I278T versus WT mice on standard diet (Fig. 7; Supplementary Tables 8 and 9). Coenzyme Q synthesis requires SAM that is dysregulated in HCU. We therefore analyzed CoQ in liver but we did not observe any significant differences in coenzyme Q content normalized for mitochondrial mass. Together, liver mitochondrial function does not seem to be impaired in transgenic I278T mouse model of HCU.

## 4 Discussion

In this study we comprehensively analyzed sulfur amino acid metabolism in mouse model of HCU in attempt to provide unified perspective into dysregulation caused by CBS deficiency and its correction by Met restriction as well as to understand possible clinical consequences of compartmentalized metabolic changes in HCU.

Metabolome represents the final level, which reflects gene and protein expression changes, functional consequences (activation, inhibitions, allosteric modulation, modification) on the expressed enzymes and proteins, resulting in fluxes of metabolites within and among organs. Our study provides new insight into tissue compartmentalization of metabolites, which has been only occasionally reported in a few selected tissues. Our main focus was on analysis of sulfur-containing and sulfur metabolism-related compounds, which in general showed the expected dysregulation reported previously in the same or other mouse models of HCU [[16], [17], [18],^22^,^49^,^50^]. Relatively small increases of Met in plasma, urine and all tissues were reported when the model was generated [18] and are in the stark contrast with clinical data, which typically find 1.5 to about 10-fold increases in plasma depending on the degree of pyridoxine responsiveness [3]. Accumulation of total Hcy, a metabolic hallmark of HCU, was observed in all body fluids and tissues of I278T mice, but mostly in plasma, urine, liver and kidney. As expected in CBS-deficient HCU, Cth concentrations were universally decreased across all compartments except heart. Another thioether Lth was similarly affected as it is a product of CBS following alternative condensation of two cysteine molecules producing H_2_S [40,41]. On the other hand, thioether Hlan, a product of condensation of two Hcy molecules, was found increased in I278T mice in all compartments. Hlan is an alternative product of CGL-catalyzed reactions [40,41] suggesting that liver and brain may have the highest expression of CGL [51].

Since activity of CBS, one of the major physiological H_2_S generators, is absent in HCU, it was expected that H_2_S concentrations as well as persulfidation would be impaired in HCU. However, we did not find substantial changes in bioavailable H_2_S in plasma of I278T mice, which is consistent with a similar observation in HCU patients [26]. However, liver H_2_S was slightly increased in I278T mice. These results leading to net zero change in plasma H_2_S could be explained by compensatory action of CGL at high levels of Hcy in HCU indicated by increased Hlan. In addition, inorganic and low-molecular-weight persulfide analysis indicated that increased levels of Hcy in HCU lead to a substantial production of HcySSH persulfide in plasma and both CysSSH persulfide and hydrogen persulfide HSS in liver, which may regulate availability of H_2_S in plasma and/or signaling in tissues [[52], [53], [54]]. A recent study using triple genetic knockout of three main enzymatic sources of H_2_S (CBS-CGL-3-MST KO mouse) showed no significant differences in reactive persulfide production compared to WT controls suggesting that perhaps CARS/CPERS system is the principal enzyme that is actually involved in and, therefore, primarily responsible for the biosynthesis of reactive persulfides and polysulfides *in vivo* in mammals [55]. Pathways and metabolites involved in H_2_S catabolism were in general neither apparently nor substantially disturbed in HCU being consistent with the data from HCU patients [26].

Cys, an end product of Hcy transsulfuration, was found substantially decreased in HCU mice being consistent with the data on humans [26]. However, urine and other tissues showed no difference between I278T and WT mice and, interestingly, liver of I278T mice showed higher concentrations compared to the WT cohort. Thus, the data suggest that plasma Cys is primarily displaced by accumulating Hcy in untreated HCU compared to the tissues, while plasma Cys is completely normalized when plasma Hcy levels were substantially decreased by Met restriction or enzyme replacement therapy [17,22]. Increased liver Cys concentration could be due to a sparing effect caused by downregulated cysteine dioxygenase (CDO) as shown in HO model of HCU [56]. Our proteomics showed a marked (∼6-fold), but non-significant downregulation of CDO. In addition, decreased liver Tau and HpT further indicated CDO downregulation. Both turnover and activity of CDO is mainly regulated by the availability of its substrate Cys with up to 45-fold increase of CDO concentration with increased protein intake and up to 10-fold increased catalytic activity in Cys excess by formation of Cys93-Tyr157 thioether-containing crosslink [57]. Unlike total Cys reported here, in the previous studies we determined non-protein-bound Cys in livers from CBS KO and I278T, which was found significantly decreased compared to WT controls [17,22]. Therefore, our observed metabolic changes could be explained by CDO sensing only available non-protein-bound Cys pool rather than total Cys. Furthermore, Cys is a rate-limiting substrate for synthesis of GSH, which was also found decreased in liver of I278T mice on a standard diet along with its precursor γ-Glu-Cys. Together, these data suggest that liver total Cys may be a misleading indicator of Cys availability in HCU.

In addition to targeted sulfur metabolome analyzed in all cohorts, we performed sphingolipidomics and determined activities of enzymes involved in oxidative phosphorylation to evaluate mitochondrial function only in the liver samples from I278T mice on a standard diet and WT controls. Previously, we and others have shown that HCU mice completely lacking CBS (CBS KO model) suffer from moderate to severe hepatopathy characterized by substantial micro- and macro-vesicular steatosis, hepatocellular necrosis with resorptive inflammatory reactions and dysregulated liver and plasma lipids manifesting in a substantially decreased fat content and lean phenotype [17,22,46]. Similar abnormalities in lipid metabolism were also identified in HCU patients [45,58]. Therefore, upregulation of sphingomyelin-ceramide-sphingosine-1-phosphate pathway in I278T mice compared to WT controls was not surprising and could indicate increased cellular stress, inflammation and cell proliferation tendencies during liver remodeling. Interestingly, we did not observe any significant changes in activities of enzymes of oxidative phosphorylation in livers of I278T mice compared to WT controls. Swollen mitochondria with disorganized cristae represent common findings in electron microscopy analysis of liver sections and biopsies from CBS knockout mice and HCU patients, respectively [17,47,48]. As it is unknown whether I278T mice manifest with swollen, disorganized mitochondria similar to the CBS KO mouse model, the reason for observing no difference in function of oxidative phosphorylation enzymes in liver in I278T versus WT mice may be due to the fact that the I278T model of HCU simply does not present this phenotype. Despite clearly disturbed glucose and lipid liver metabolism, the plasma markers of liver function, such as alanine and aspartate aminotransferases and alkaline phosphatase, were previously determined to be similar to those of negative controls [22]. Similarly, in terms of oxidative stress markers, we previously found the activities of liver superoxide dismutase and catalase in plasma slightly elevated indicating the presence of oxidative stress [22]. Alternatively, the functional consequences may not be apparent in the selected, individually assayed enzymes of oxidative phosphorylation pathway, but rather at the mitochondrial respiration as a whole.

Dysregulated metabolism is often a consequence of changes at gene expression as well as protein expression and regulation levels. The I278T mice showed substantially disturbed liver proteome compared to WT controls, while Met restrictions normalized most of the dysregulated proteins. Several cytochrome P450 proteins (CYPs) were upregulated, such as CYP4A14, CYP4A10, CYP2A5, CYP2C37 and CYP2D10, while only a single CYP3A41 and CYP2D70 were found downregulated (Supplementary Tables 3 and 4). The CYP4A14 (mouse ortholog of human CYP4A22) catalyzes ω-hydroxylation of medium-chain fatty acids and arachidonic acid and is involved in lipid metabolism. Specifically, ablation of CYP4A14 attenuated hepatic inflammation, steatosis and fibrosis in mice [59]. The CYP4A10 (mouse ortholog of human CYP4A11) catalyzes ω-hydroxylation of long-chain fatty acids, particularly dodecanoic (lauric) acid in liver [60]. The CYP2A5 (mouse ortholog of human CYP2A6) was found upregulated under various conditions causing liver injury, such as nicotine/smoke exposure, liver cancer, hepatic infection or heavy metals and solvent exposure, and tightly regulated by nuclear factor erythroid 2-like 2 (Nrf2), a transcription factor activated under redox stress [61]. The CYP2C37 (mouse ortholog of human CYP2C19) catalyzes ω-hydroxylation of arachidonic acid producing 12-hydroxyeicosatetraenoic acid (12-HETE) [62]. Previous analysis of I278T liver transcriptome by DNA microarrays showed downregulation of stearoyl-CoA desaturase-1 (SCD1), a key enzyme in lipogenesis, which correlated with the lean phenotype of I278T mice [46] and was also confirmed by our proteomic analysis presented here. We speculate that upregulation of CYPs involved in fatty acid oxidations is a response to catabolic stress resulting in an increased utilization of fatty acids for energy production in I278T consequently leading to a depleted liver fat storage [22] supported by downregulation of SCD1 and lean phenotype. Related to the increased expression of several CYPs, it was interesting to observe upregulation of epoxide hydrolase 1 (EPHX1) in I278T mice. EPHX1 hydrolyzes epoxyeicosanoids (EETs), which have, among many other, anti-inflammatory effects, into biologically less active dihydroxyeicosatrienoic acids (DHETs) [63]. Furthermore, expression of EPHX1 is activated by the redox stress-responsive transcription factor Nrf2 likely to promote detoxification of highly reactive epoxides into less reactive diols [64]. We speculate that decrease of anti-inflammatory EETs by upregulated EPHX1 and increase of pro-inflammatory HETEs by upregulated CYPs, particularly CYP2C37, result in a pro-inflammatory environment in liver affecting through these metabolites cardiovascular system and other organs in I278T mice. Indeed, previously we found significant upregulation of IL-12 (p40), IL-13 and TNF-α pro-inflammatory cytokines in plasma of I278T mice [22].

Two major detoxification pathways were found upregulated in I278T mice: the UDP-glucuronidation and S-glutathionylation (Supplementary Tables 3 and 4). Upregulation of seven UDP-glucuronosyltransferases UGT1A1, UGT1A6, UGT1A6B, UGT2B34, UGT2B35, UGT2B36 and UGT3A was supported by upregulation of UDP-glucose-6-dehydrogenase UGDH, which converts UDP-glucose into UDP-glucuronate [65]. Six glutathione-S-transferases were found upregulated in liver of I278T mice: GSTA4, GSTM1, GSTM3, GSTM4, GSTO1 and GSTT2. All are involved in detoxification of various toxins, including products of oxidative damage, by conjugation with GSH. GSTA4 is particularly interesting as it detoxifies 4-hydroxynonenal, a product of arachidonic acid peroxidation and a biomarker of oxidative tissue damage [66]. Similarly to the case of glucuronidation, activity of GST enzymes was supported by upregulation of both the catalytic and regulatory subunits of gamma-glutamate-cysteine ligase, a rate-limiting enzyme in biosynthesis of GSH, and of glutathione synthetase [67]. Furthermore, the most upregulated protein in liver of I278T mice was asparagine synthetase (ASNS), which catalyzes conversion of aspartate and glutamine into asparagine and glutamate [68], likely to provide glutamate for GSH biosynthesis. Upregulation of ASNS is typically mediated by the redox regulator Nrf2 [69]. The second most upregulated protein in I278T liver was phosphoserine aminotransferase 1 (PSAT1) catalyzing conversion of 3-phosphohydroxypyruvate into phosphoserine using glutamate as a donor of amino group forming α-ketoglutarate. PSAT1 is a target of ATF4 regulation in *de novo* Ser/Gly synthesis pathway [69] and plays an important regulatory role in tumorigenesis and malignant progression [70,71]. Specifically, inhibition of PSAT1 reduced biosynthesis of GSH, promoted redox stress, DNA damage and apoptosis. Taken together, we hypothesize that these protein expression changes indicate increased effort of I278T hepatocytes to produce glutamate and glycine, which in turn would support biosynthesis of GSH to mitigate cellular stress and the damage caused by oxidative stress and lipid peroxidation. Dysregulated protein expression and subsequent metabolic response and adaptation seem to be mediated by simultaneous activation of Nrf2 and ATF4 to potentiate the expression of cytoprotective genes and upregulate GSH biosynthesis under the stress conditions caused by CBS deficiency.

Previously we found that increased Met intake in I278T mice resulted in a fatal intestinal bleeding and identified by both RNAseq and ELISA Met intake-dependent downregulation of liver-synthesized coagulation factor XI (F11) [72]. Our proteomic analysis confirmed downregulation of this target as well (Supplementary Table 4). HCU patients are susceptible to thromboembolic events [9], but I278T mice do not show pro-thrombotic phenotype. Therefore, Sikora et al. [73] compared plasma proteomics to identify molecular basis for this discrepancy. They did not find F11 to be downregulated in plasma of I278T mice, but the coagulation system as a whole was strongly affected by CBS deficiency in HCU patients.

Our study offered a unique insight into the efficacy of MRD within tissues. Interestingly, severe Met restriction led to a substantial decrease of plasma, urine and liver Met, but essentially normalization in other tissues suggesting that the most metabolically “exposed” compartments are plasma, urine and liver. As expected in CBS-deficient HCU, Cth concentrations were universally decreased across all compartments except heart, and further lowered by MRD. On the other hand, thioether Hlan, a product of condensation of two Hcy molecules by CGL [40,41], was found increased in I278T mice in all compartments. MRD caused its massive decrease in liver and brain with the remaining tissues being less responsive or almost insensitive, such as kidney. Interestingly, the highest expression of CGL was found in mouse liver and brain [51], which, in turn, would be affected most by substantially decreased availability of Hcy as a result of treatment of HCU with MRD. The I278T mice showed substantially disturbed liver proteome compared to WT controls; however, dietary Met restriction normalized most of the dysregulated proteins, thus indicating a profound beneficial effect of MRD on protein expression in HCU.

In conclusion, the presented data helped us understand and summarize the dysregulation caused by CBS deficiency in HCU and how this dysregulated system is affected by Met restriction, a current cornerstone therapy for pyridoxine non-responsive HCU. We showed that metabolic changes in plasma and urine, the only readily available samples in clinics, do not necessarily reflect the situation in tissues. Interestingly, CBS deficiency did not cause substantial overall changes in H_2_S concentrations, but pointed towards the role of differential persulfidation potentially involved in signal transduction.

## Funding

TM acknowledges support from the 10.13039/501100005869University of Fribourg (Research Pool grant 22-15) and the 10.13039/100000001Swiss National Science Foundation (project funding 10.001.133). VK was supported by the grants 19-08786S (10.13039/501100001824Czech Science Foundation), and institutional programs RVO-10.13039/501100016366VFN 64165 (10.13039/501100016366General University Hospital in Prague) and Cooperatio – Metabolic Disorders (10.13039/100007397Charles University). JP and OV were supported by Cooperatio – BIOLOGY (10.13039/100007397Charles University) and National Institute for Cancer Research (No. LX22NPO5102) funded by the 10.13039/100006939EU, Programme EXCELES. HH was supported by institutional programs Cooperatio in Paediatrics and UNCE 24/MED/022 (10.13039/100007397Charles University). LK was supported by the institutional programs of 10.13039/100007397Charles University in Prague (UNCE/MED/007 and Cooperatio/DIAG/1FM). TA was supported in part by Grant-in-Aid for Transformative Research Areas, International Leading Research, and Scientific Research (Challenging Exploratory Research) from the Ministry of Education, Culture, Sports, Sciences and Technology (10.13039/501100001700MEXT), Japan (21H05258, 21H05263, 23K20040, and 22K19397); by 10.13039/501100002241Japan Science and Technology Agency (JST), Japan, 10.13039/501100003382CREST Grant Number JPMJCR2024; and by a grant from the 10.13039/100009619Japan Agency for Medical Research and Development (10.13039/100009619AMED). WDK acknowledges support from 10.13039/100000002National Institutes of Health, USA (R01DK101404). PN and TD acknowledge support from the National Research, Development and Innovation Fund of the Ministry of Culture and Innovation under the National Laboratories Program (National Tumor Biology Laboratory (2022–2.1.1-NL-2022-00010) and the Hungarian Thematic Excellence Program (project TKP2021-EGA-44) Grant Agreements with the National Research, Development and Innovation Office. PN has received funding from the HUN-REN Hungarian Research Network (grant 1500207).

## CRediT authorship contribution statement

**Tomas Majtan:** Writing – review & editing, Writing – original draft, Supervision, Investigation, Funding acquisition, Conceptualization. **Thomas Olsen:** Writing – review & editing, Visualization, Software, Methodology, Investigation, Funding acquisition, Formal analysis, Data curation. **Jitka Sokolova:** Investigation, Formal analysis. **Jakub Krijt:** Writing – review & editing, Methodology, Investigation. **Michaela Křížková:** Investigation. **Tomoaki Ida:** Investigation. **Tamás Ditrói:** Writing – review & editing, Methodology, Investigation. **Hana Hansikova:** Writing – review & editing, Methodology, Investigation, Funding acquisition. **Ondrej Vit:** Investigation. **Jiri Petrak:** Writing – review & editing, Methodology, Investigation, Funding acquisition. **Ladislav Kuchař:** Writing – review & editing, Investigation, Funding acquisition. **Warren D. Kruger:** Writing – review & editing, Resources, Funding acquisition. **Péter Nagy:** Writing – review & editing, Funding acquisition. **Takaaki Akaike:** Writing – review & editing, Supervision, Methodology, Funding acquisition. **Viktor Kožich:** Writing – review & editing, Writing – original draft, Supervision, Funding acquisition, Formal analysis, Conceptualization.

## Declaration of competing interest

The authors declare no conflict of interests.

## Data Availability

The data are either available in the manuscript or from a public repository as referenced within the manuscript.
